# Supplementary material for: Mohangic Acid H and Mohangiol: New p-Aminoacetophenone Derivatives from a Mudflat-Derived Streptomyces sp
Source: Mar Drugs. 2025 Jul 30;23(8):307. doi: 10.3390/md23080307 (PMC12387680; doi:10.3390/md23080307)
Supplement: Supplementary file 1 [file marinedrugs-23-00307-s001.zip › marinedrugs-3746100-supplementary.pdf]

Supplementary Materials

Mohangic acid H and Mohangiol: New *p*-Aminoacetophenone Derivatives from a Mudflat-Derived *Streptomyces* sp.

Juwan Son [1], Ju Heon Lee [1], Yong-Joon Cho [2, 3], Kyuho Moon [4, *], Munhyung Bae [1, *]

1 College of Pharmacy, Gachon University, Incheon 21936, Republic of Korea

2 Department of Molecular Bioscience, Kangwon National University, Chuncheon 24341, Republic of Korea

3 Multidimensional Genomics Research Center, Kangwon National University, Chuncheon 24341, Republic of Korea

4 College of Pharmacy, Kyung Hee University, Seoul 02447, Republic of Korea

Corresponding Author

* Correspondence: kmoon@khu.ac.kr, Tel.: +82-2-961-2139 (K.M); baemoon89@gachon.ac.kr, Tel.: +82-31-820-4921 (M.H.)

Table of contents

Figure S1. (**A**) The bacterial strain *Streptomyces* sp. AWH31-250 on YEME agar, (**B**) Chemical profiles of culture of *Streptomyces* sp. AWH31-250, (**C**) Sampling site, (**D**) UV and MS spectra of mohangic acid H (**1**) and mohangiol (**2**).
Figure S2. ^{1}H NMR spectrum (600 MHz) of mohangic acid H (**1**) in pyridine-d_5.
Figure S3. ^{13}C NMR spectrum (150 MHz) of mohangic acid H (**1**) in pyridine-d_5.
Figure S4. COSY spectrum (600 MHz) of mohangic acid H (**1**) in pyridine-d_5.
Figure S5. HSQC spectrum (600 MHz) of mohangic acid H (**1**) in pyridine-d_5.
Figure S6. HMBC NMR spectrum (600 MHz) of mohangic acid H (**1**) in pyridine-d_5.
Figure S7. ROESY spectrum (600 MHz) of mohangic acid H (**1**) in pyridine-d_5.
Figure S8. ^{1}H NMR spectrum (600 MHz) of mohangiol (**2**) in pyridine-d_5.
Figure S9. ^{13}C NMR spectrum (150 MHz) of mohangiol (**2**) in pyridine-d_5.
Figure S10. COSY spectrum (600 MHz) of mohangiol (**2**) in pyridine-d_5.
Figure S11. HSQC spectrum (600 MHz) of mohangiol (**2**) in pyridine-d_5.
Figure S12. HMBC spectrum (600 MHz) of mohangiol (**2**) in pyridine-d_5.
Figure S13. ROESY spectrum (600 MHz) of mohangiol (**2**) in pyridine-d_5.
Figure S14. Optimized geometries of conformers of diastereomer 2a (2*R*) of mohangiol (**2**).
Figure S15. Optimized geometries of conformers of diastereomer 2b (2*S*) of mohangiol (**2**).
Figure S16. Isolation schemes of mohangic acid H (**1**) and mohangiol (**2**) from *Streptomyces* sp. AWH31-250.
Figure S17. HR-ESIMS spectrum of (**a**) mohangic acid H (**1**), (**b**) mohangiol (**2**), and (**c**) mohangic acid A.
Figure S18. Genome map of *Streptomyces* sp. AWH31-250.
Figure S19. The 16s rRNA sequence of *Streptomyces* sp. AWH31-250 (1399 nt).
Figure S20. Maximum-likelihood tree based on 16s rRNA sequence of *Streptomyces* sp. AWH31-250.
Figure S21. Comparative sequence alignment of ketoreductase (KR) domains from *mohB-C*, *FscB-C*, highlighting conserved fingerprint motifs 1-10.
Table S1. Relative potential energy-MMFF94 of conformers found in **Figure 3**.
Table S2. Boltzmann population of conformers found in **Figure 3**.
Table S3. Experimental and calculated chemical shifts of mohangiol (**2**).
Table S4. antiSMASH results table of *Streptomyces* sp. AWH31-250.
Table S5. Deduced putative functions of ORFs in **1**, **2** and candicidin biosynthetic gene cluster from region 2.1 of *Streptomyces* sp. AWH31-250.
Table S6. Summary of stereospecificity-determining motifs in *β*-module KRs: motifs highlighted in red denote primary motifs, while the remaining motifs serve as secondary support.

Table S7. Amino acid sequences of ketoreductase (KR) of *mohB*, *mohC* and their homologs *FscB* and *FscC*.
Table S8. Amino acid sequences of representative *β*-module ketoreductase (KR) subtypes.
Table S9. Comparative sequence alignment of ketoreductase (KR) domains from *mohB*, *mohC* and their homologs *FscB* and *FscC*.
Table S10. Results of antibacterial assay of **1** and **2**.
Table S11. Results of antifungal assay of **1** and **2**.

Figure S1. (**A**) The bacterial strain *Streptomyces* sp. AWH31-250 on YEME agar, (**B**) Chemical profiles of culture of *Streptomyces* sp. AWH31-250, (**C**) Sampling site, (**D**) UV and MS spectra of mohangic acid H (**1**) and mohangiol (**2**).

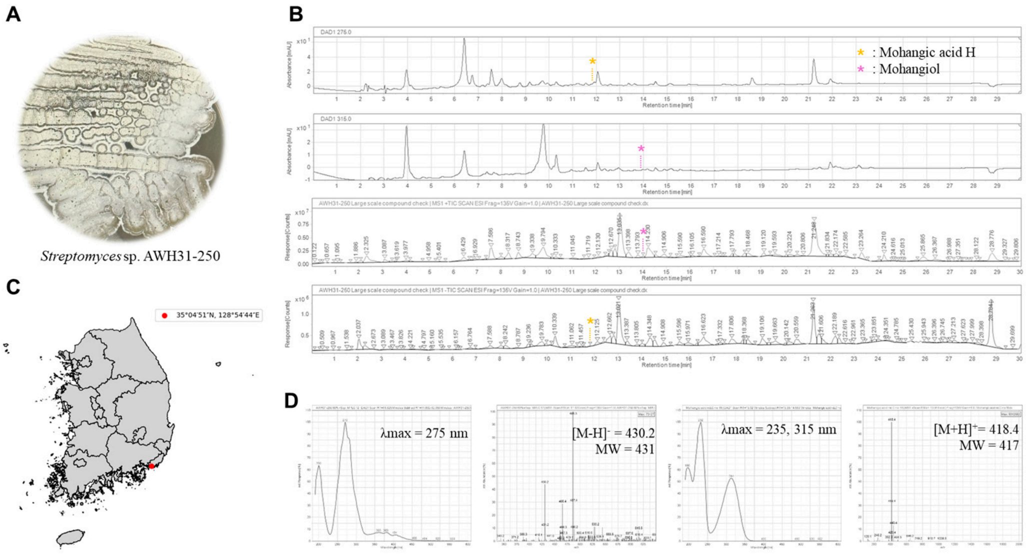

* (**C**) was sourced from the GADM (Global Administrative Areas) database (version 4.1), available at https://gadm.org/.

Figure S2. ^1H NMR spectrum (600 MHz) of mohangic acid H (**1**) in pyridine-d_5.

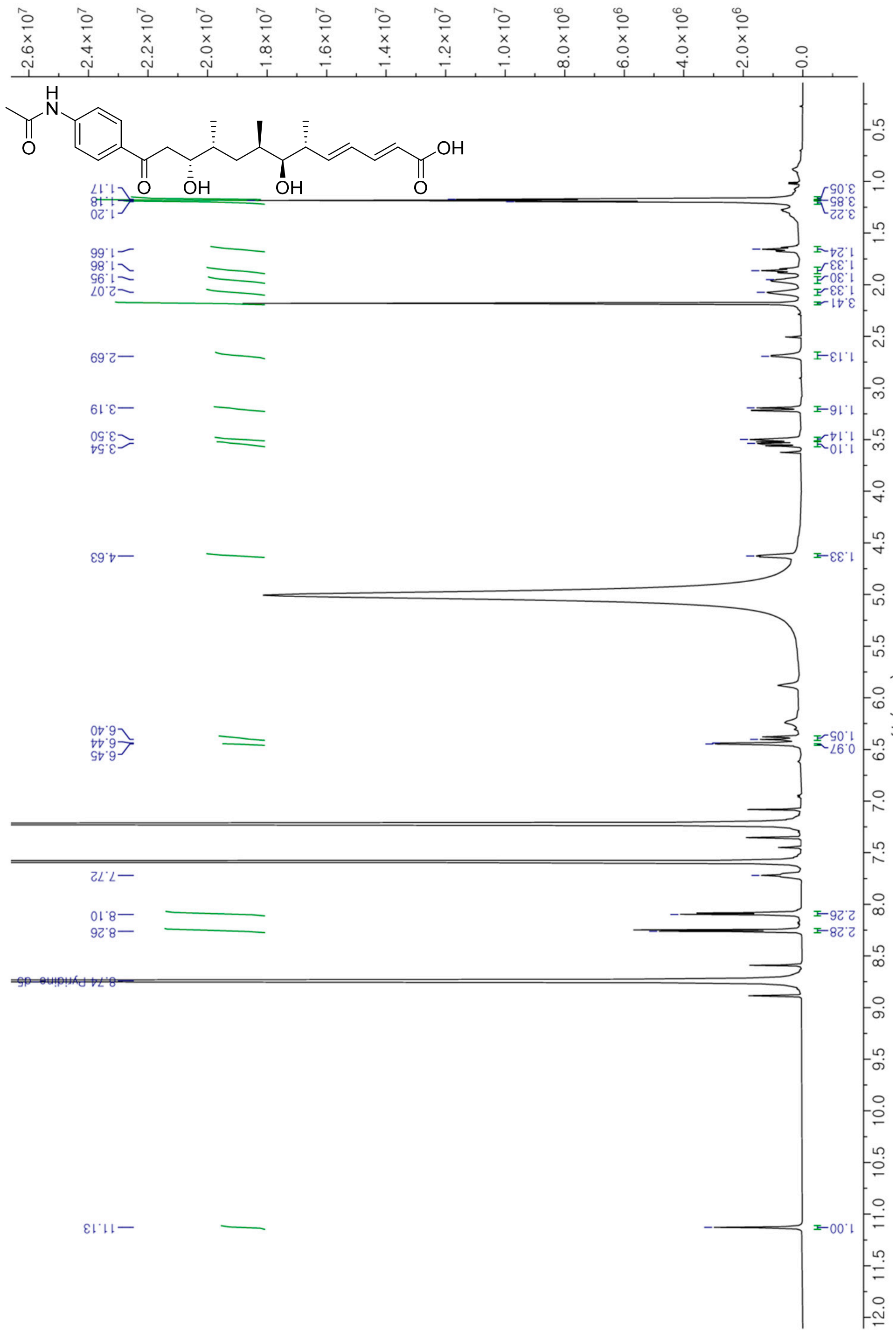

Figure S3. ^{13}C NMR spectrum (150 MHz) of mohangic acid H (**1**) in pyridine-d_5.

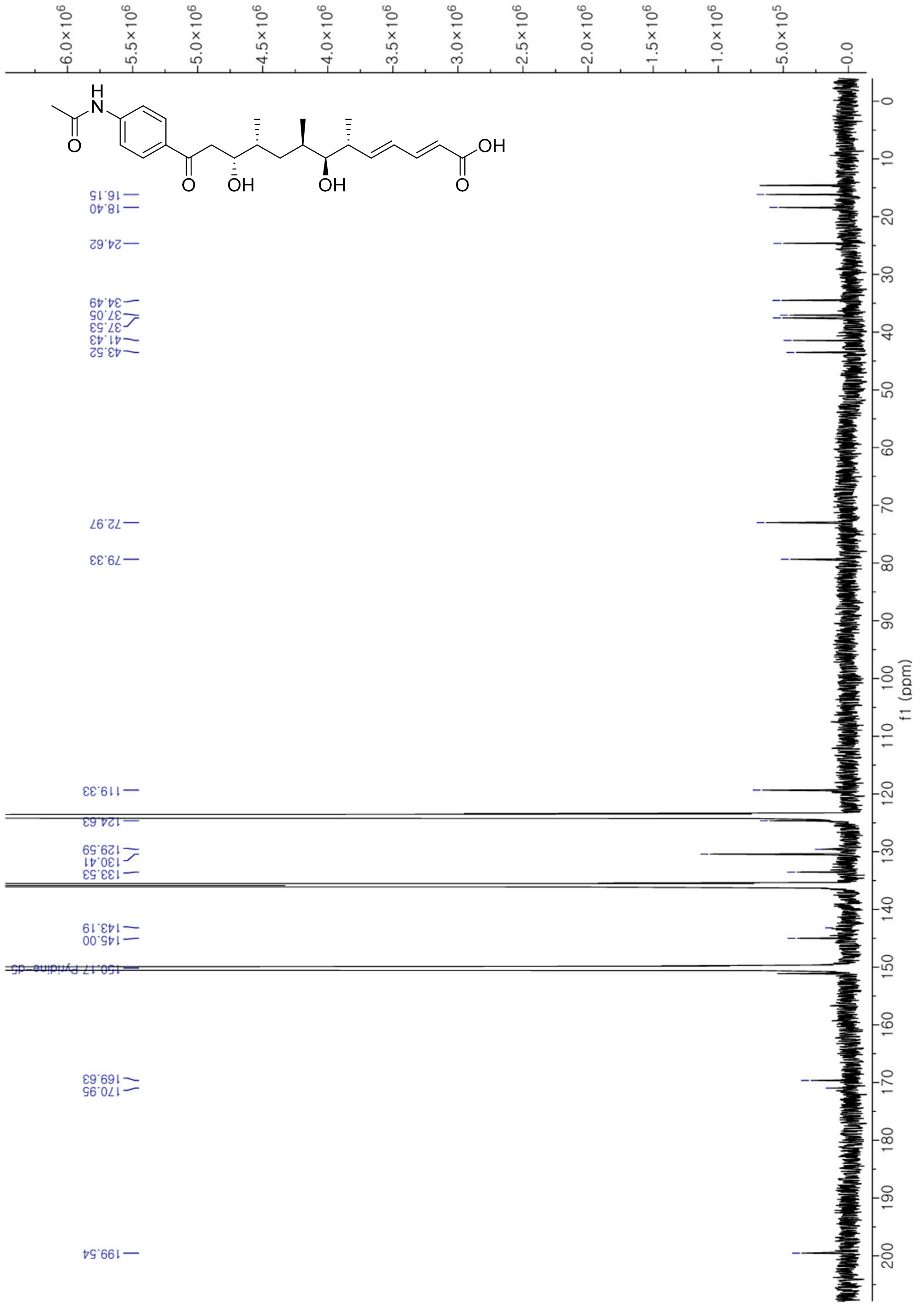

Figure S4. COSY spectrum (600 MHz) of mohangic acid H (**1**) in pyridine-d_5.

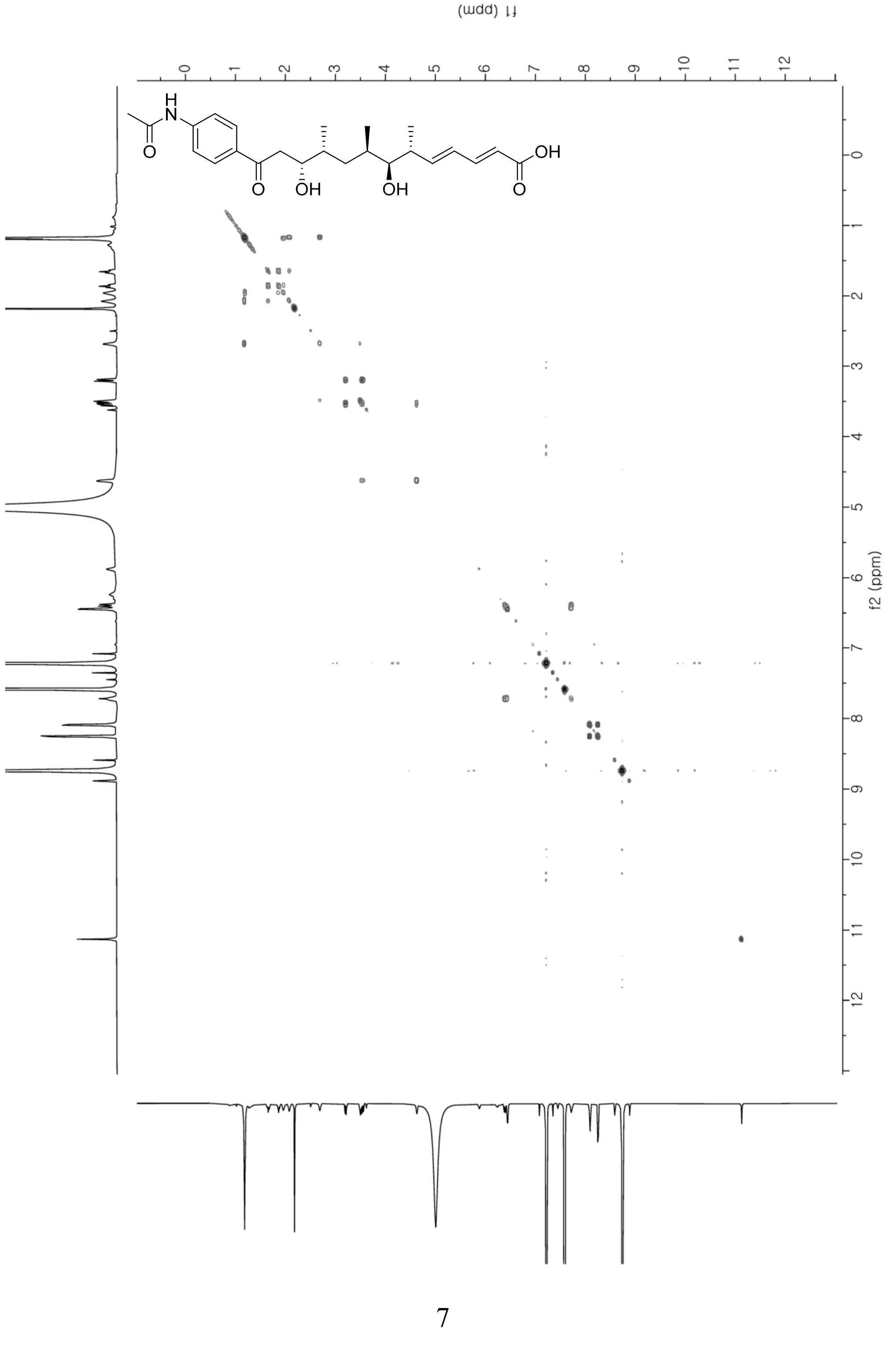

Figure S5. HSQC spectrum (600 MHz) of mohangic acid H (**1**) in pyridine-d_5.

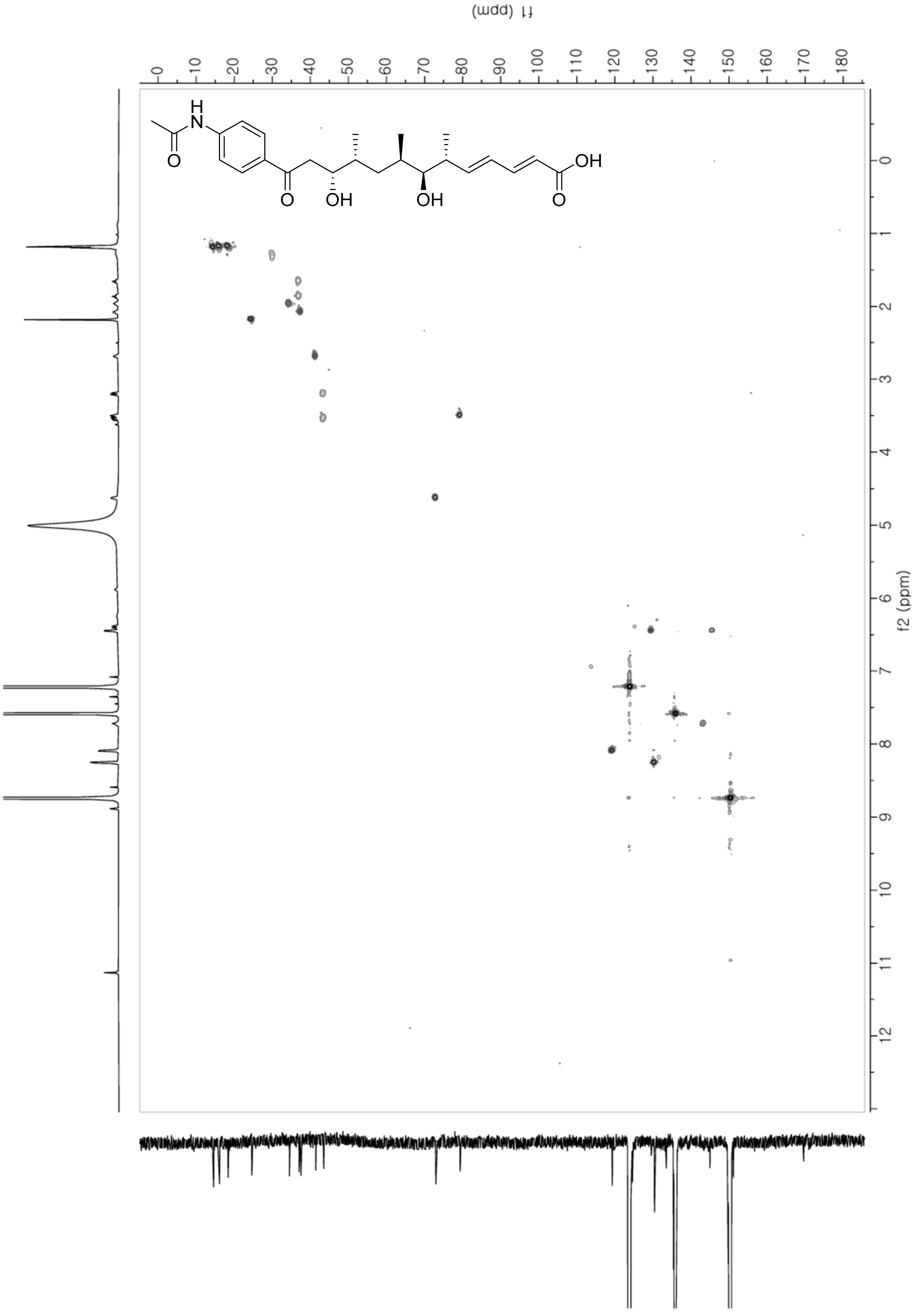

Figure S6. HMBC spectrum (600 MHz) of mohangic acid H (**1**) in pyridine-d_5.

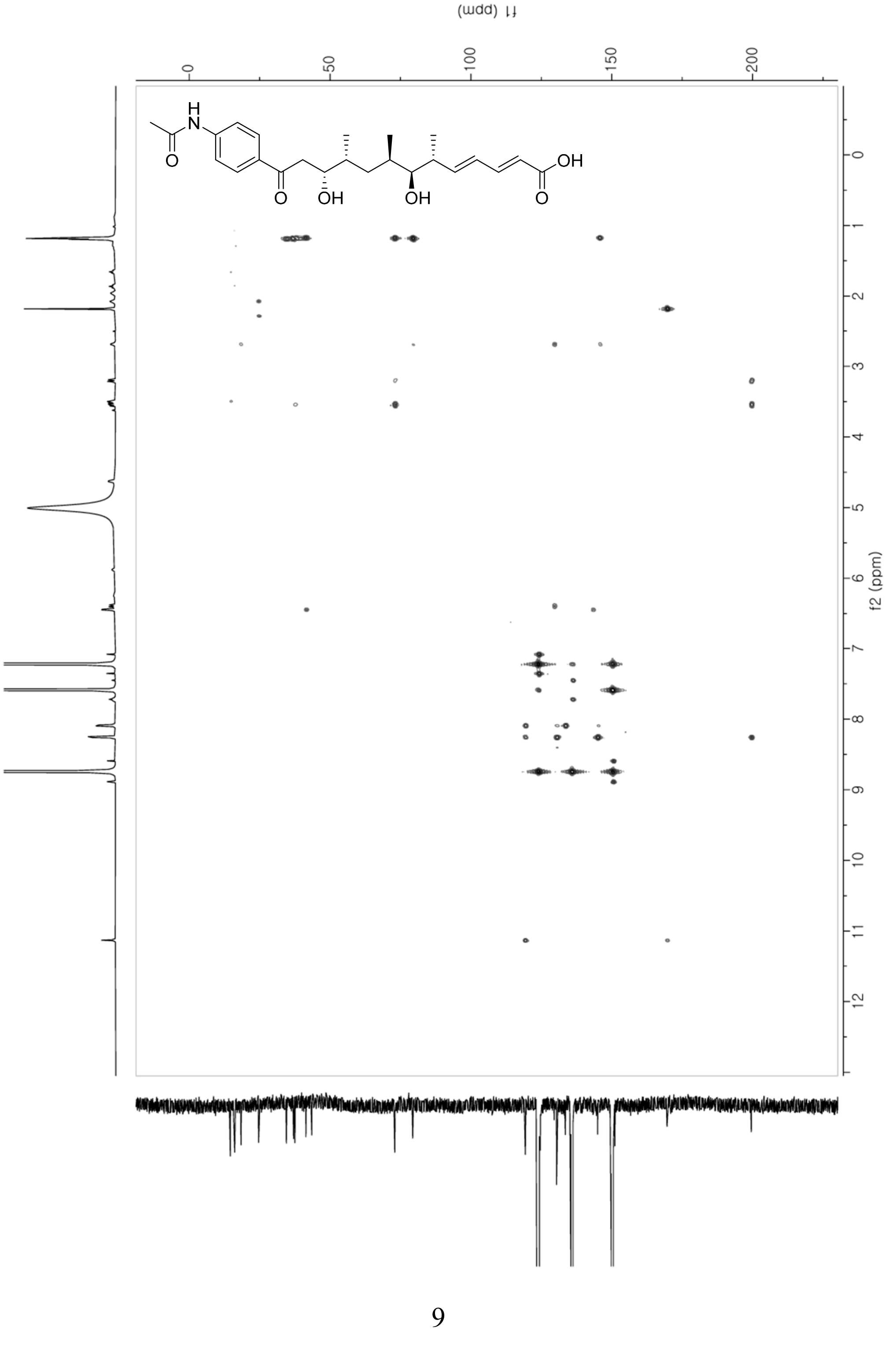

Figure S7. ROESY spectrum (600 MHz) of mohangic acid H (**1**) in pyridine-*d*5.

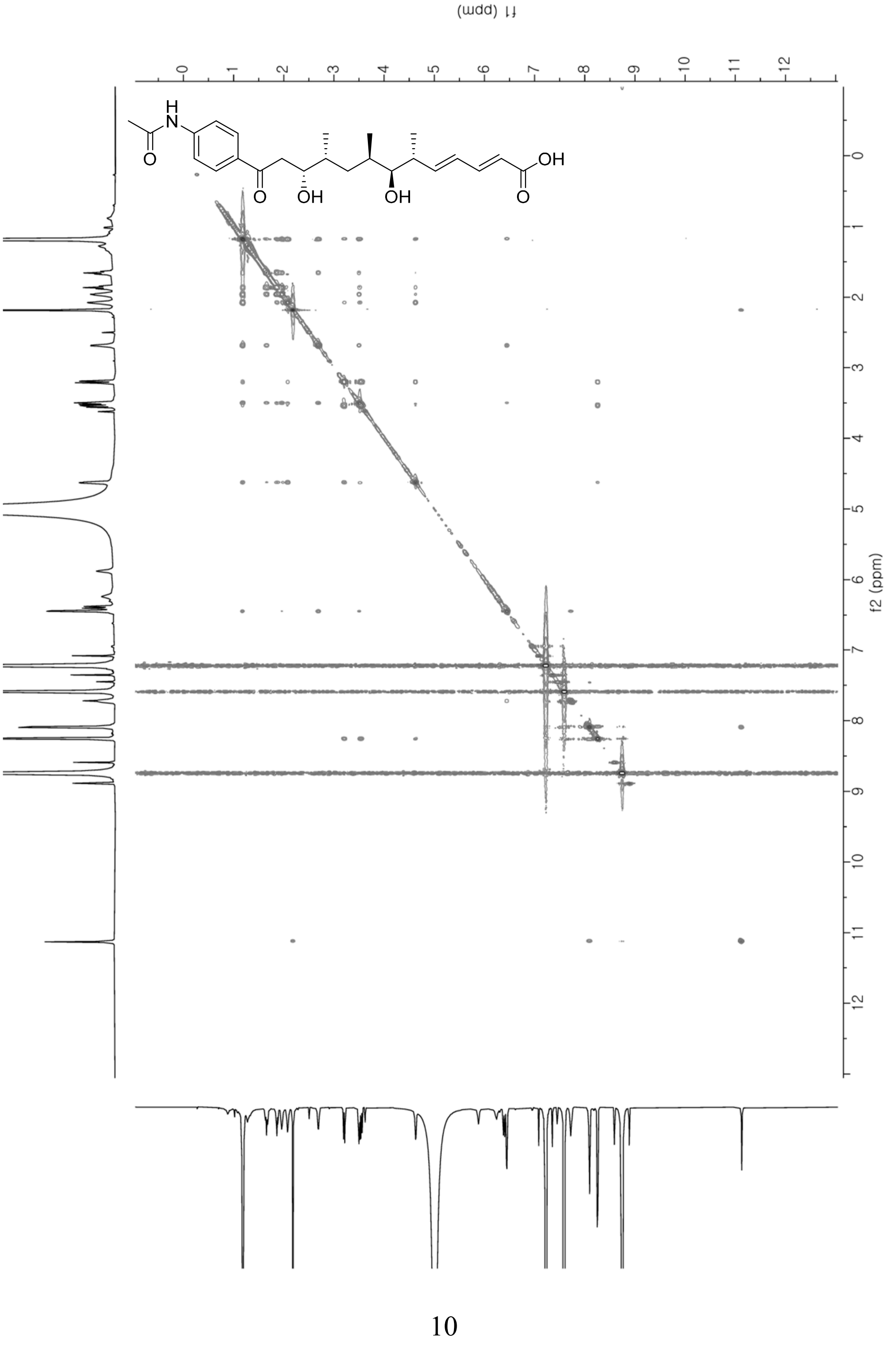

Figure S8. ^{1}H NMR spectrum (600 MHz) of mohangiol (**2**) in pyridine-*d*5.

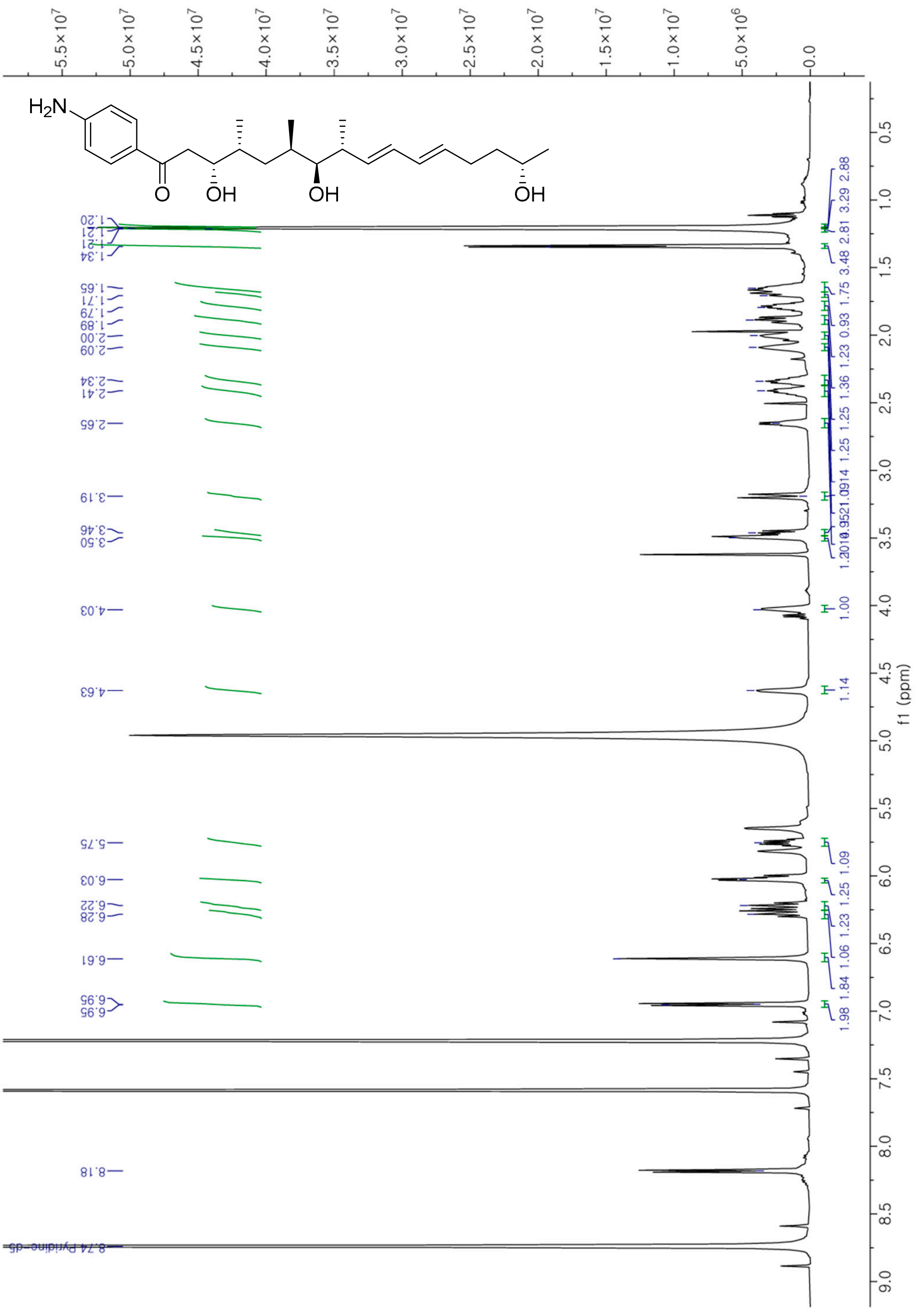

Figure S9. ^{13}C NMR spectrum (150 MHz) of mohangiol (**2**) in pyridine-d_5.

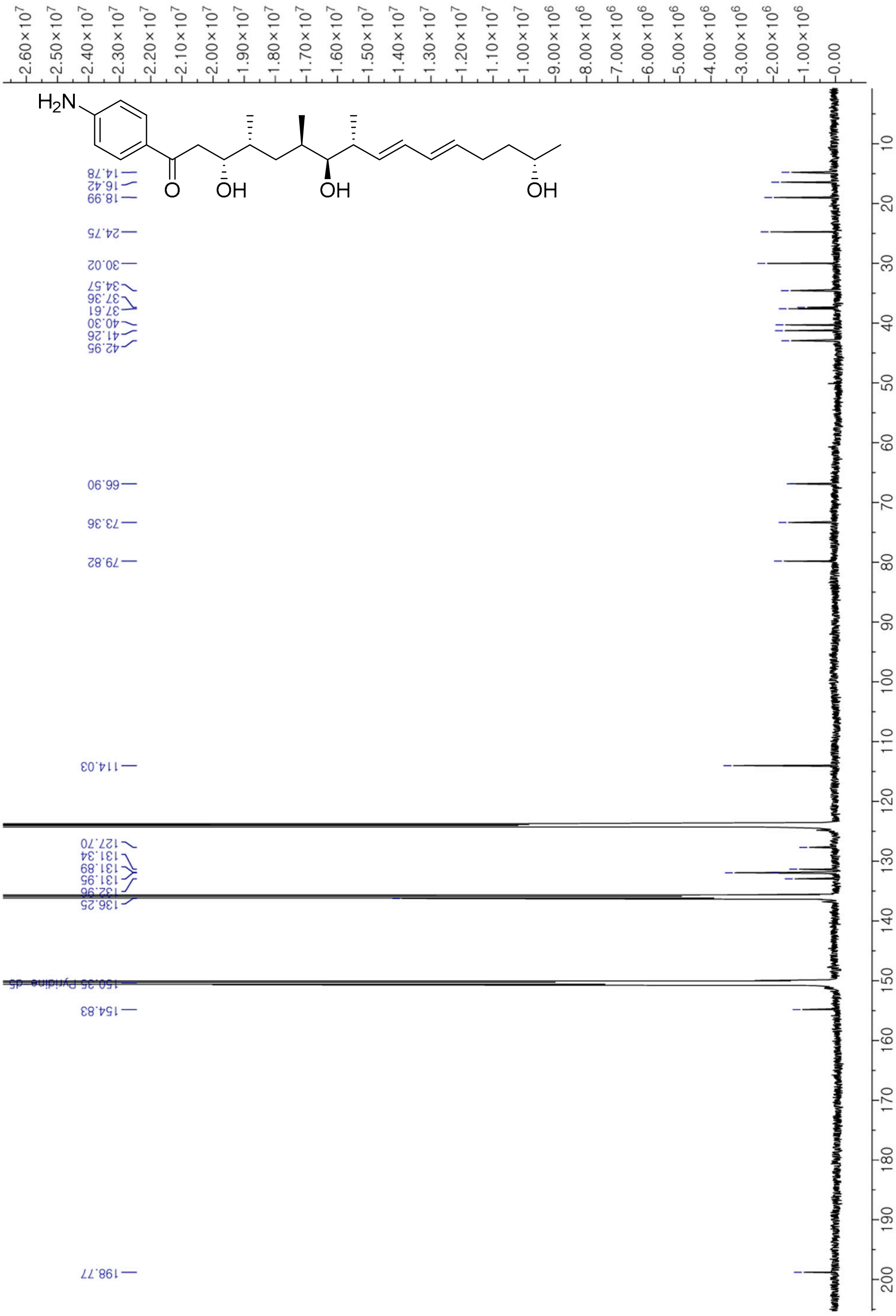

Figure S10. COSY spectrum (600 MHz) of mohangiol (**2**) in pyridine-d_5.

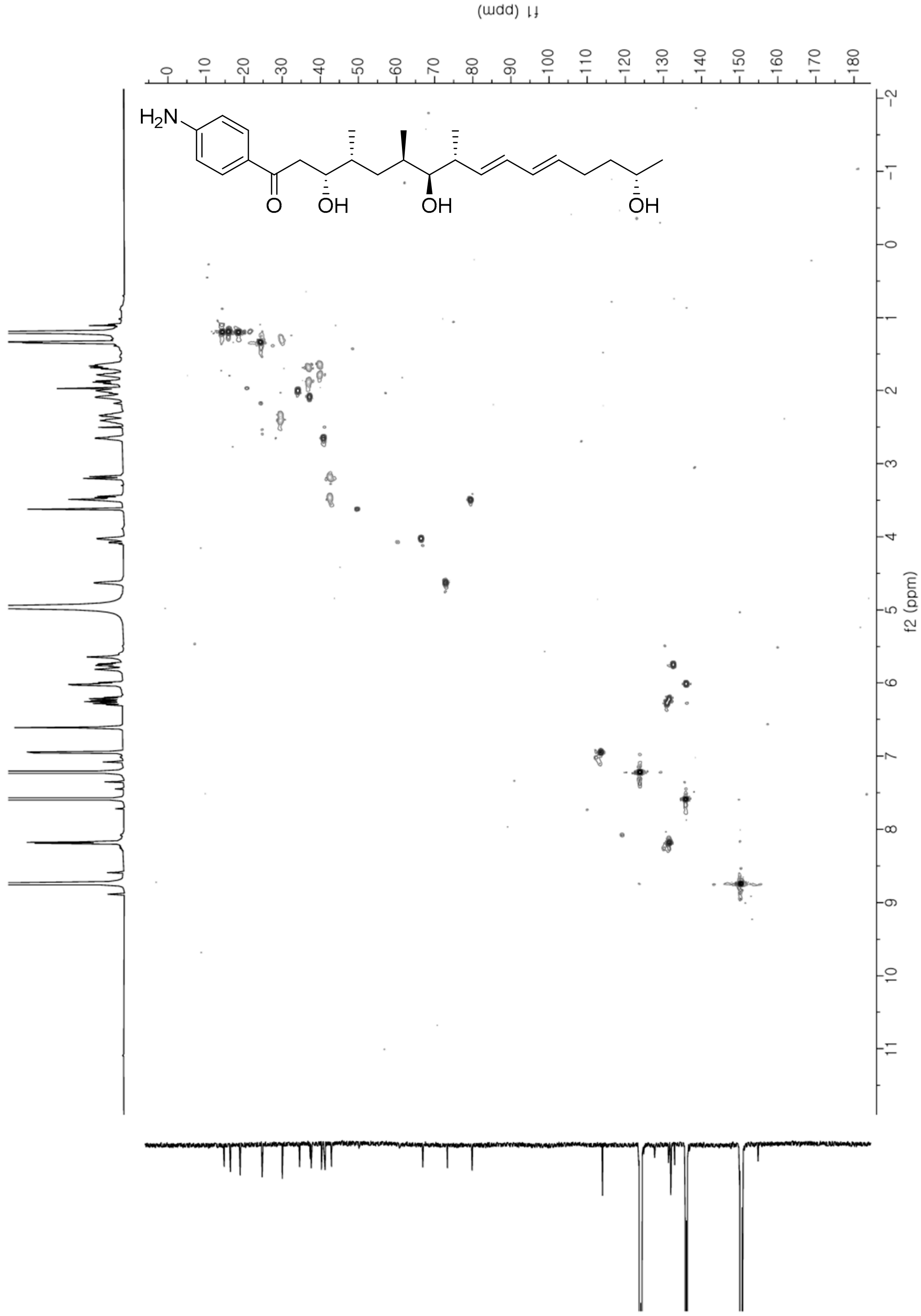

Figure S11. HSQC spectrum (600 MHz) of mohangiol (**2**) in pyridine-d_5.

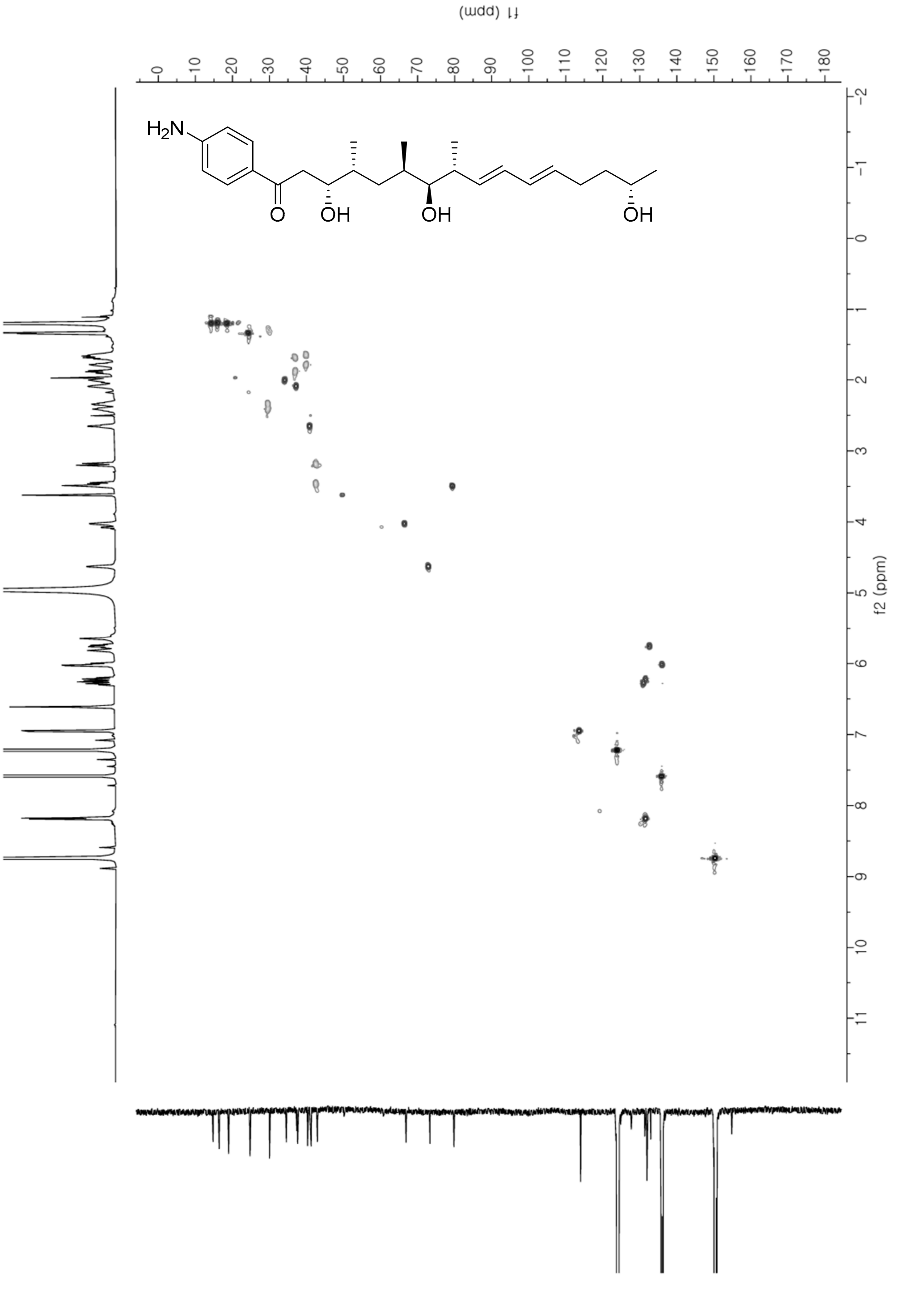

Figure S12. HMBC spectrum (600 MHz) of mohangiol (**2**) in pyridine-d_5.

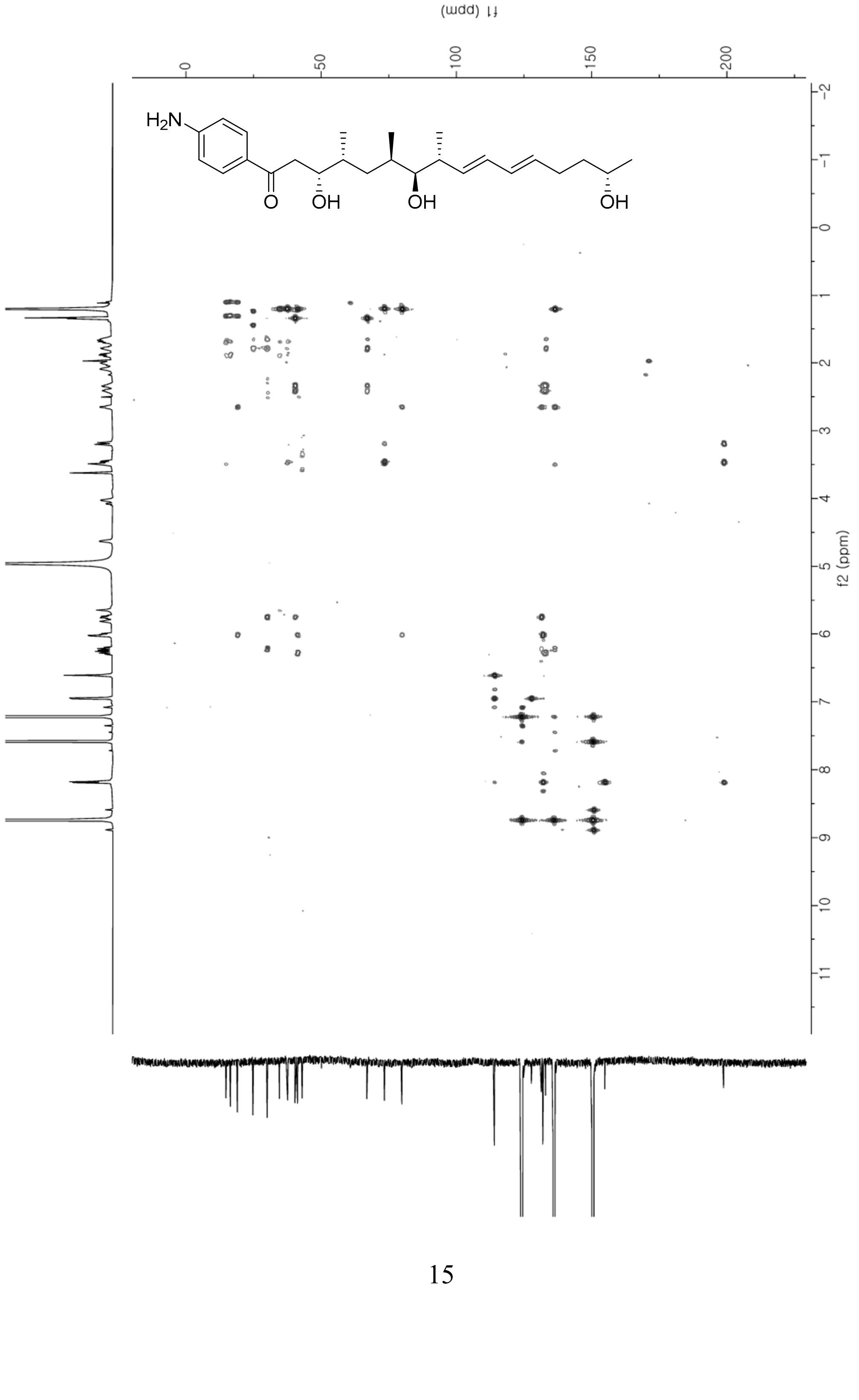

Figure S13. ROESY spectrum (600 MHz) of mohangiol (**2**) in pyridine-d_5.

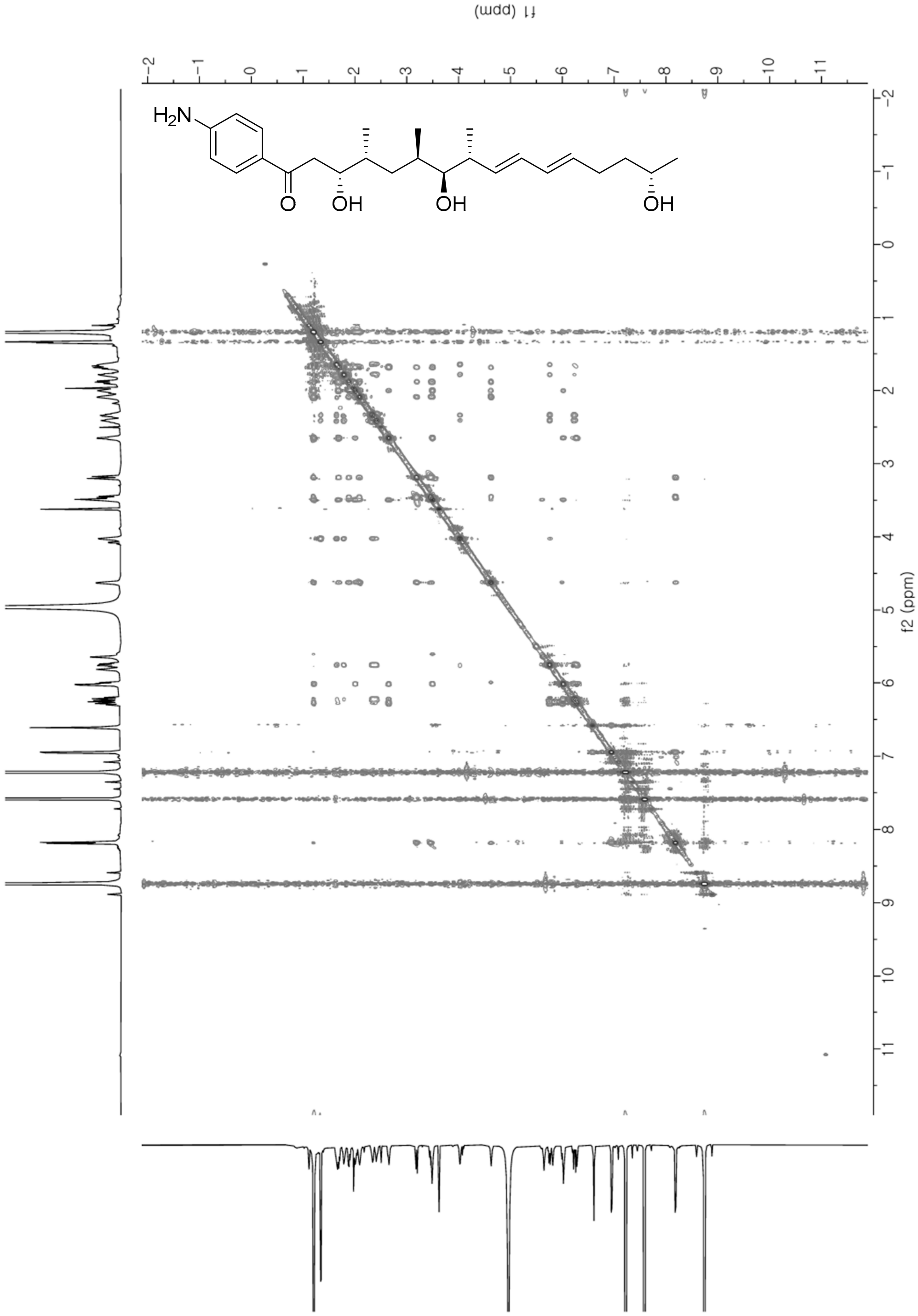

Table S1. Relative potential energy-MMFF94 of conformers found in **Figure 3**.

**Diastereomer 2a (2*R*)**		**Diastereomer 2b (2*S*)**	
	Relative Potential Energy-MMFF94		Relative Potential Energy-MMFF94
Conformer 1	0.000 kJ/mol	Conformer 1	0.000 kJ/mol
Conformer 2	7.643 kJ/mol	Conformer 2	4.148 kJ/mol
Conformer 3	8.757 kJ/mol	Conformer 3	5.698 kJ/mol
Conformer 4	10.264 kJ/mol	Conformer 4	6.018 kJ/mol
		Conformer 5	6.110 kJ/mol

Table S2. Boltzmann population of conformers found in **Figure 3**.

**Diastereomer 2a (2*R*)**		**Diastereomer 2b (2*S*)**	
	Boltzmann Population		Boltzmann Population
Conformer 1	87.110%	Conformer 1	48.883%
Conformer 2	3.990%	Conformer 2	9.172%
Conformer 3	2.545%	Conformer 3	4.908%
Conformer 4	1.386%	Conformer 4	4.313%
		Conformer 5	4.156%

Table S3. Experimental and calculated chemical shifts of mohangiol (**2**).

Position	Experimental chemical shifts	Calculated chemical shifts of 2a (*R*)	Calculated chemical shifts of 2b (*S*)
C-1	24.7	28.4	27.8
C-2	66.9	63.6	66.2
C-3	40.3	41.5	42.0
C-4	30.0	32.5	33.4
C-5	132.9	135.2	132.6
C-6	131.8	131.3	134.2
C-7	131.3	132.2	131.0
C-8	136.2	136.1	141.4
C-9	41.2	46.24	48.0
C-10	79.8	84.0	76.2
C-11	34.5	38.0	35.5
C-12	37.3	32.8	41.2
C-13	37.6	38.8	39.9
C-14	73.3	77.6	72.6
C-15	42.9	45.8	46.2
C-16	198.7	207.6	201.4
C-17	18.9	22.0	19.4
C-18	14.7	18.7	17.7
C-19	16.4	17.3	18.3
C-1′	127.7	135	128.3
C-2′	131.9	130.6	132.8
C-3′	114.0	115.4	115.2
C-4′	154.8	144.6	148.4
C-5′	114.0	115.9	113.6
C-6′	131.9	129.8	131.5
H_3-1a	1.35	0.52	0.93
H_3-1b	1.35	0.58	1.44
H_3-1c	1.35	0.88	1.18
H-2	4.05	2.83	3.94
H_2-3a	1.64	1.4	1.56
H_2-3b	1.79	0.97	1.21
H_2-4a	2.34	2.58	2.21
H_2-4b	2.41	1.77	2.4
H-5	5.75	5.96	5.69
H-6	6.22	6.12	6.43
H-7	6.28	6.23	6.13

H-8	6.03	5.97	5.17
H-9	2.65	2.31	2
H-10	3.49	3.22	3.26
H-11	2	1.52	1.77
H_2-12a	1.70	1.14	1.57
H_2-12b	1.88	1.33	1.58
H-13	2.09	2	2.04
H-14	4.63	4.08	3.91
H_2-15a	3.19	2.97	3.18
H_2-15b	3.46	2.75	2.32
H_3-17a	1.21	1.16	0.74
H_3-17b	1.21	0.89	1.11
H_3-17c	1.21	1.33	0.73
H_3-18a	1.2	0.4	1.22
H_3-18b	1.2	1.79	0.84
H_3-18c	1.2	1.08	0.85
H_3-19a	1.21	1.13	0.88
H_3-19b	1.21	0.44	1.21
H_3-19c	1.21	0.5	0.98
H-2′	8.18	7.84	8.27
H-3′	6.95	6.83	6.71
H-5′	6.95	6.99	6.56
H-6′	8.18	7.87	7.79

Figure S14. Optimized geometries of conformers of diastereomer **2a** (2*R*) of mohangiol (**2**).

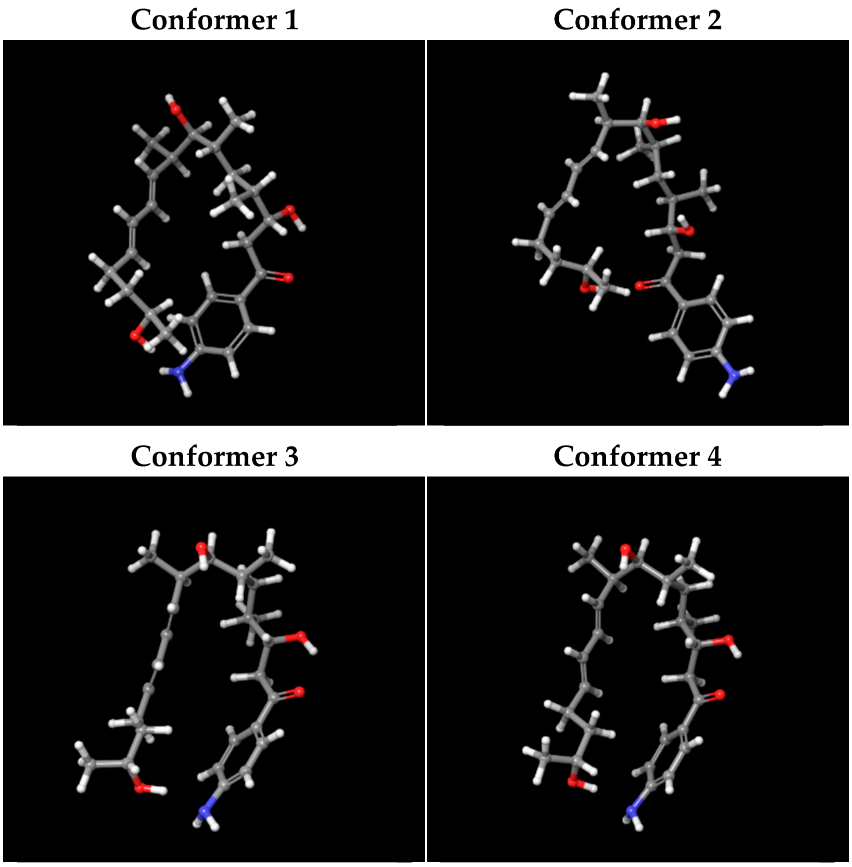

Figure S15. Optimized geometries of conformers of diastereomer **2b** (2*S*) of mohangiol (**2**).

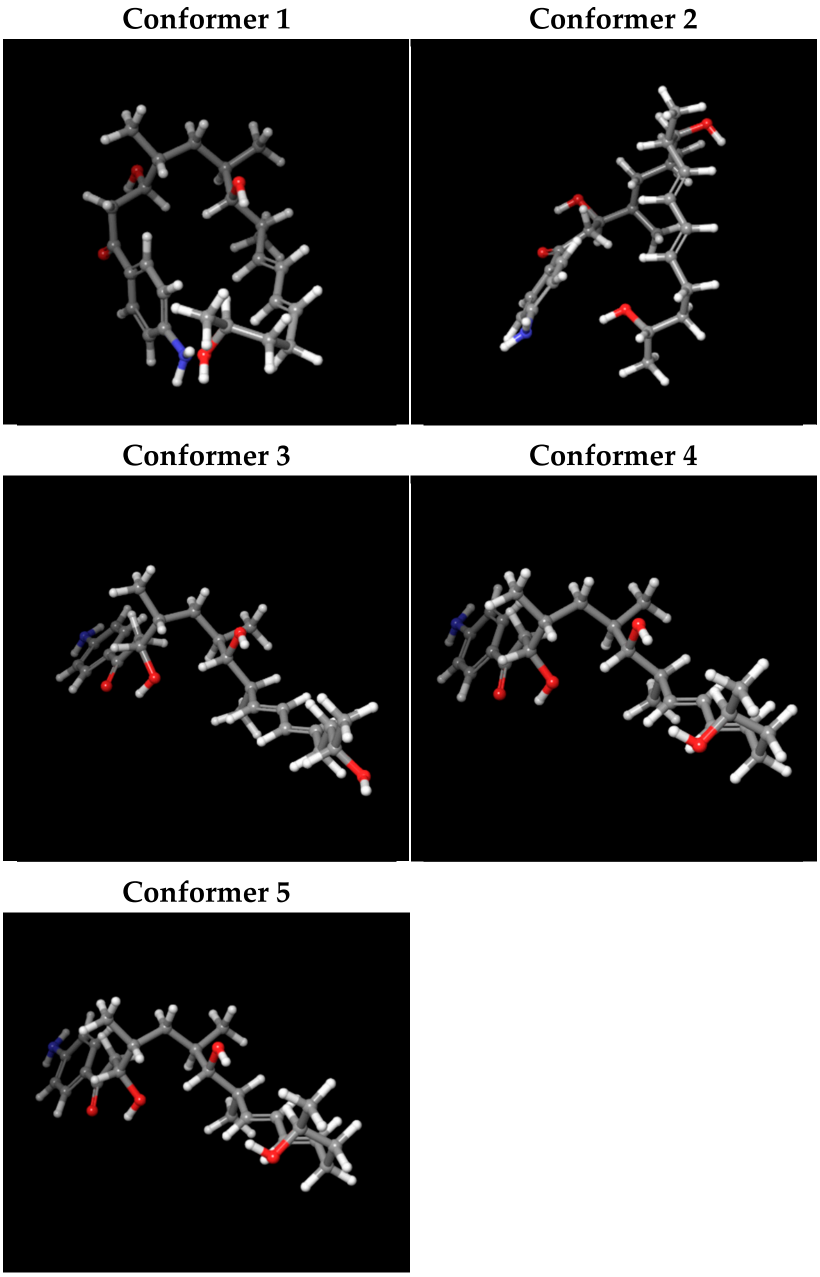

Figure S16. Isolation schemes of mohangic acid H (**1**) and mohangiol (**2**) from *Streptomyces* sp. AWH31-250.

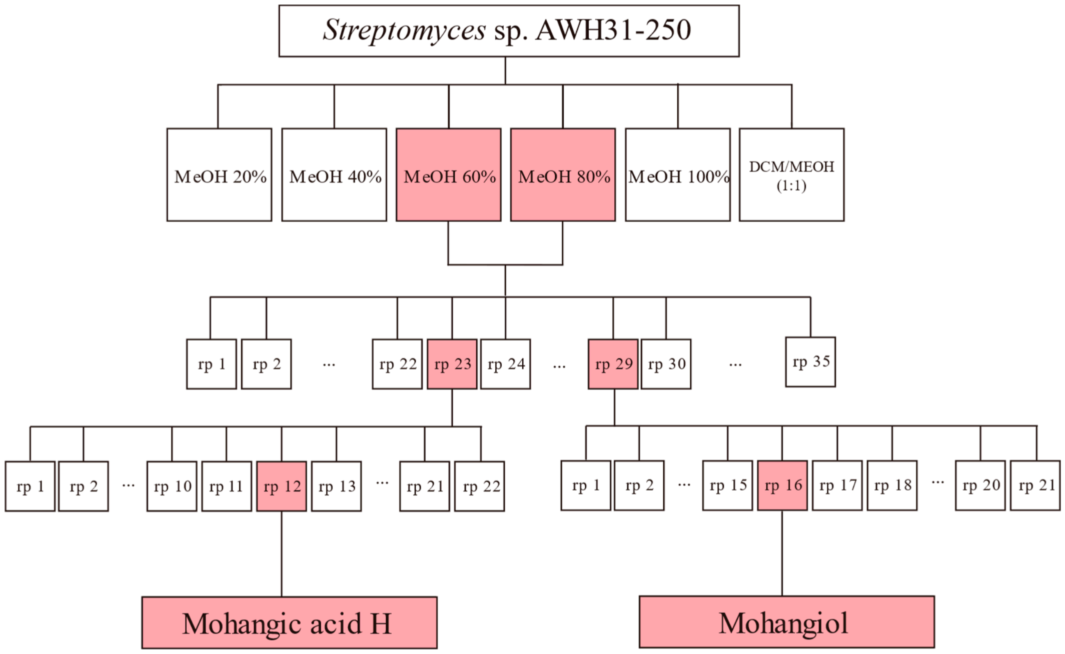

Figure S17. HR-ESIMS spectrum of (a) mohangic acid H (**1**), (b) mohangiol (**2**), and (c) mohangic acid A.

(a)

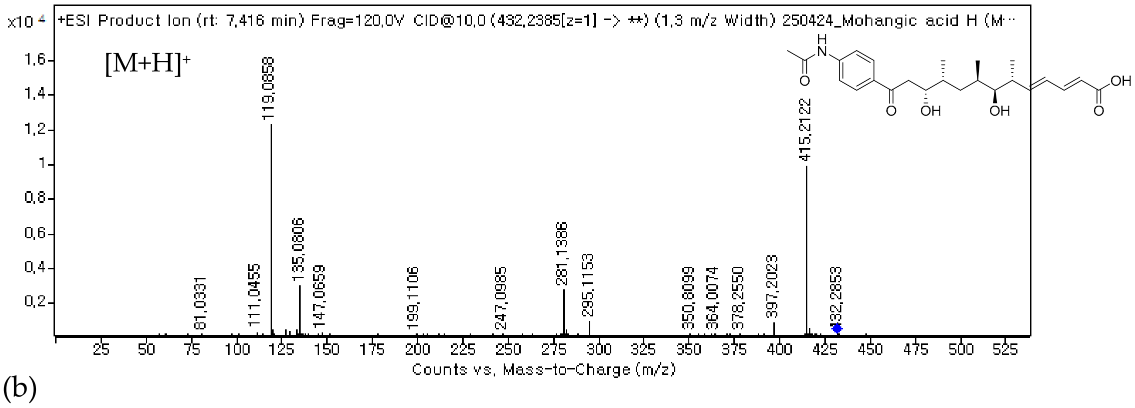

(b)

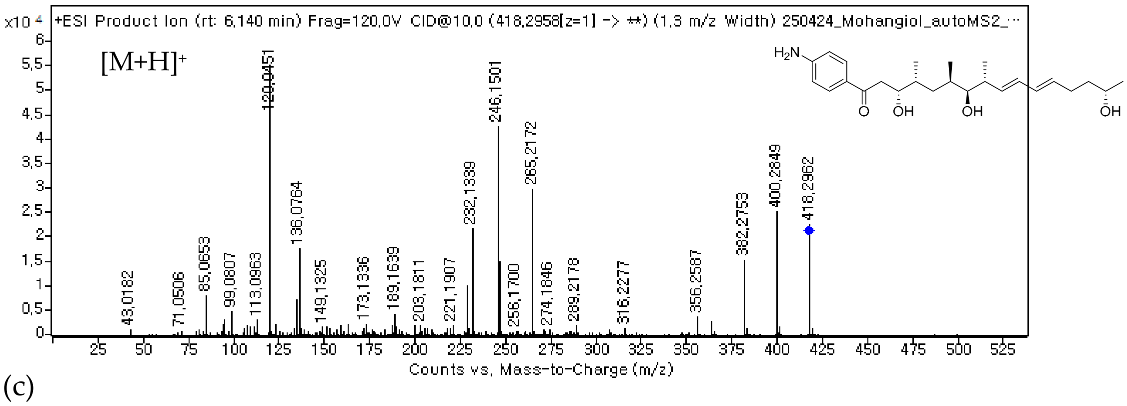

(c)

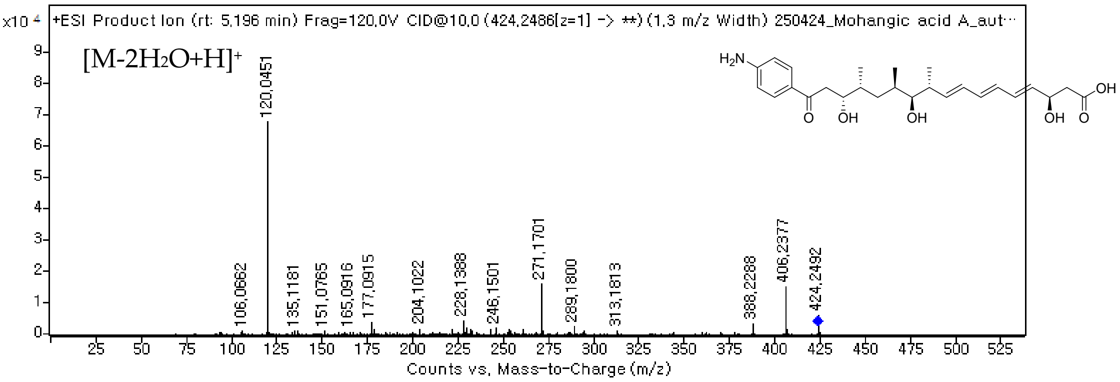

Figure S18. Genome map of *Streptomyces* sp. AWH31-250.

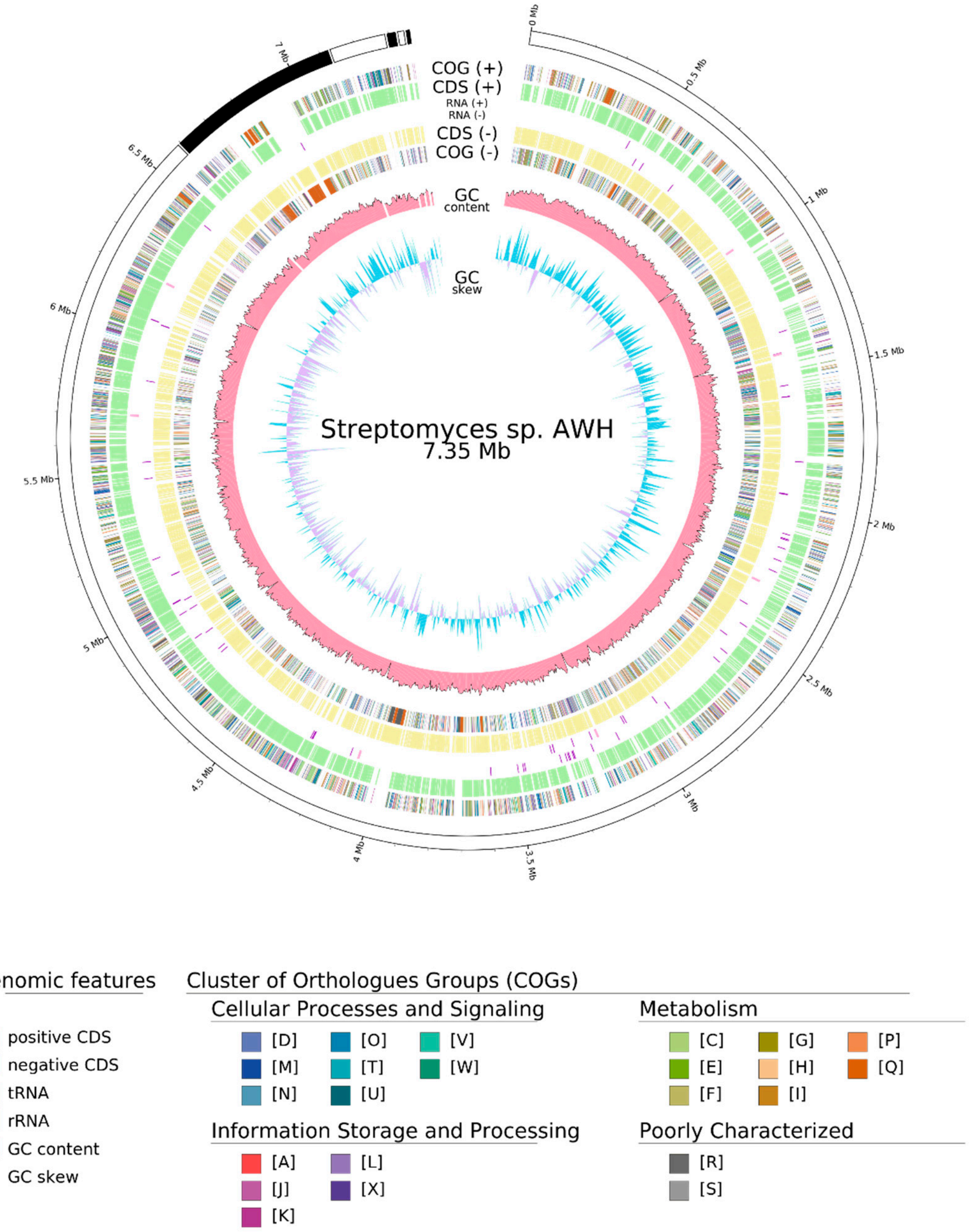

Figure S19. The 16s rRNA sequence of *Streptomyces* sp. AWH31-250 (1399 nt).

TGCAGTCGAACGATGAACCGCTTTCGGGCGGGGATTAGTGGCGAACGGGTGA
GTAACACGTGGGCAATCTGCCCTGCACTCTGGGACAAGCCCTGGAAACGGGG
TCTAATACCGGATATGACCGTCTGCCGCATGGTGGATGGTGTAAAGCTCCGGCG
GTGCAGGATGAGCCCGCGGCCTATCAGCTTGTTGGTGAGGTAGTGGCTCACCA
AGGCGACGACGGGTAGCCGGCCTGAGAGGGCGACCGGCCACACTGGGACTG
AGACACGGCCCAGACTCCTACGGGAGGCAGCAGTGGGGAATATTGCACAATG
GGCGAAAGCCTGATGCAGCGACGCCGCGTGAGGGATGACGGCCTTCGGGTTG
TAAACCTCTTTCAGCAGGGAAGAAGCGAAAGTGACGGTACCTGCAGAAGAAG
CGCCGGCTAACTACGTGCCAGCAGCCGCGGTAATACGTAGGGCGCAAGCGTT
GTCCGGAATTATTGGGCGTAAAGAGCTCGTAGGCGGCTTGTCACGTCGGTTGT
GAAAGCCCGGGGCTTAACCCCGGGTCTGCAGTCGATACGGGCAGGCTAGAGT
TCGGTAGGGGAGATCGGAATTCCTGGTGTAGCGGTGAAATGCGCAGATATCAG
GAGGAACACCGGTGGCGAAGGCGGATCTCTGGGCCGATACTGACGCTGAGGA
GCGAAAGCGTGGGGAGCGAACAGGATTAGATACCCTGGTAGTCCACGCCGTA
AACGGTGGGCACTAGGTGTGGGCAACATTCCACGTTGTCCGTGCCGCAGCTA
ACGCATTAAGTGCCCCGCCTGGGGAGTACGGCCGCAAGGCTAAAACTCAAAG
GAATTGACGGGGGCCCGCACAAGCGGCGGAGCATGTGGCTTAATTCGACGCA
ACGCGAAGAACCTTACCAAGGCTTGACATACACCGGAAACGTCTGGAGACAG
GCGCCCCCTTGTGGTCGGTGTACAGGTGGTGCATGGCTGTCGTCAGCTCGTGT
CGTGAGATGTTGGGTTAAGTCCCGCAACGAGCGCAACCCTTGTCCCGTGTTGC
CAGCAGGCCCTTGTGGTGCTGGGGACTCACGGGAGACCGCCGGGGTCAACTC
GGAGGAAGGTGGGGACGACGTCAAGTCATCATGCCCCTTATGTCTTGGGCTGC
ACACGTGCTACAATGGCCGGTACAATGAGCTGCGATACCGCGAGGTGGAGCG
AATCTCAAAAAGCCGGTCTCAGTTCGGATTGGGGTCTGCAACTCGACCCCATG
AAGTCGGAGTCGCTAGTAATCGCAGATCAGCATTGCTGCGGTGAATACGTTCC
CGGGCCTTGTACACACCGCCCGTCACGTCACGAAAGTCGGTAACACCCGAAG
CCGGTGGCCCAACCCCTTGTGGGAGGGAGCTGTCGAAG

Figure S20. Maximum-likelihood tree based on 16s rRNA sequence of *Streptomyces* sp. AWH31-250.

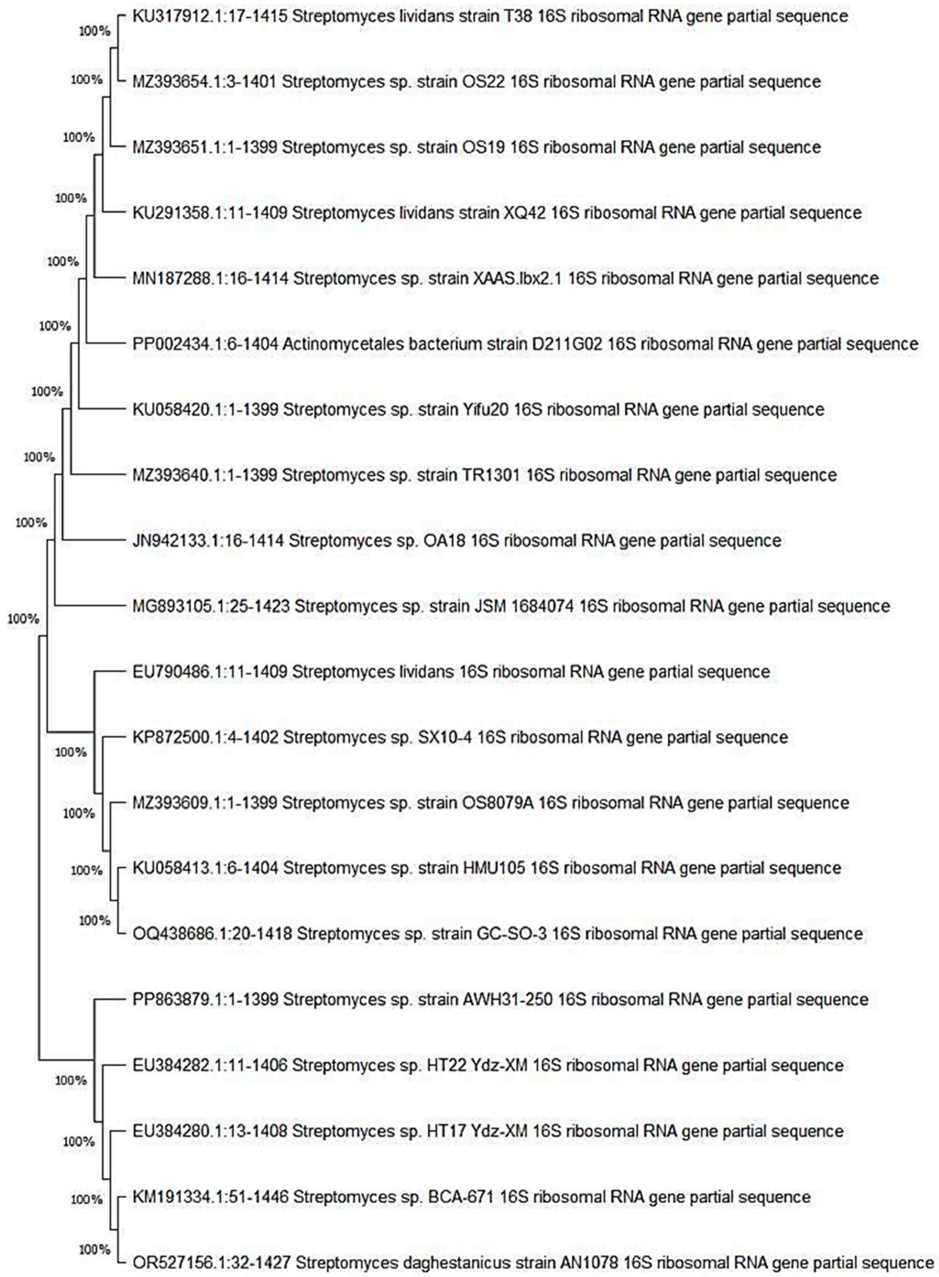

Figure S21. Comparative sequence alignment of ketoreductase (KR) domains from *mohB-C*, *FscB-C*, highlighting conserved fingerprint motifs 1-10.

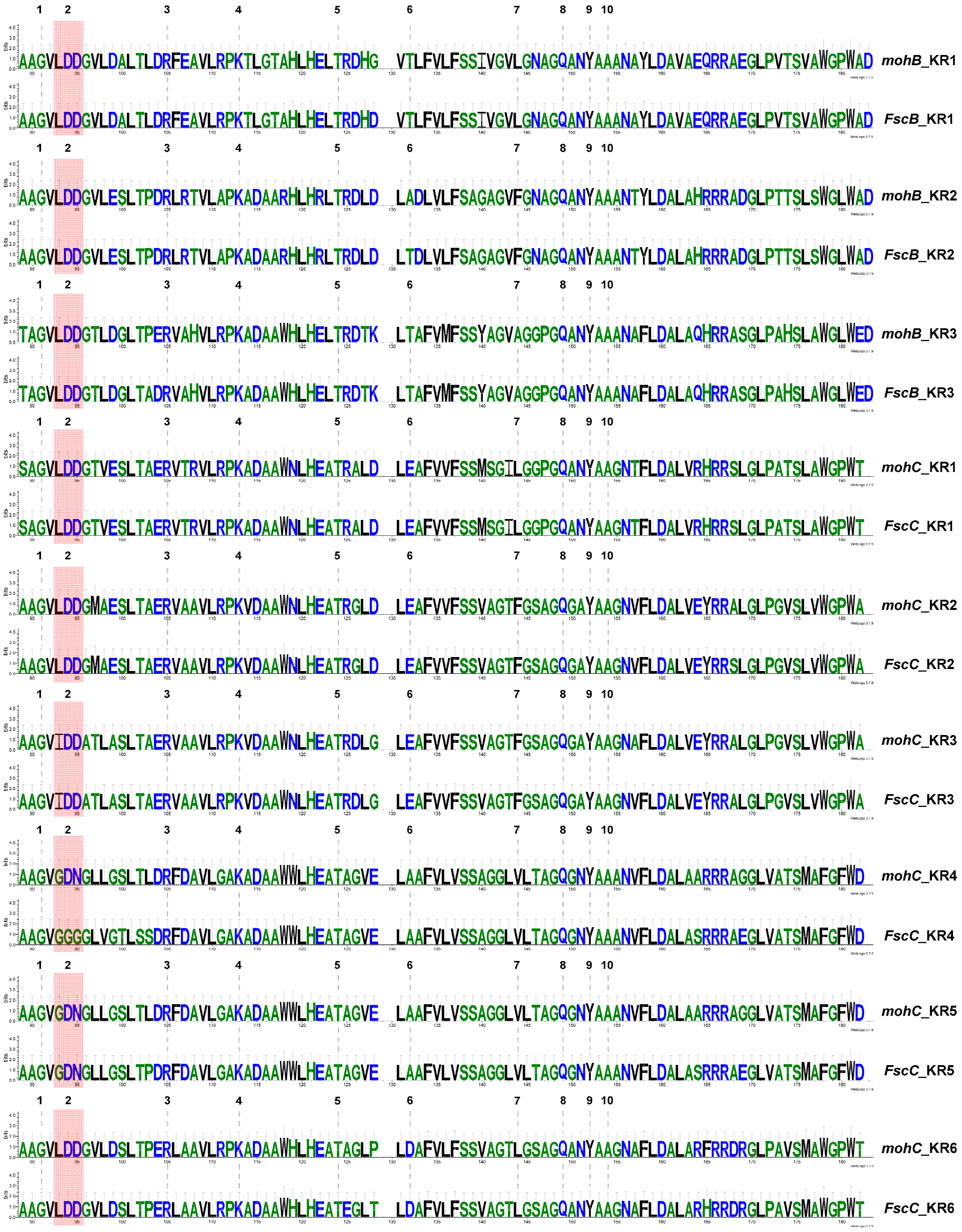

Table S4. antiSMASH results table of *Streptomyces* sp. AWH31-250.

Region	Type	From	To	Most similar known cluster	Similarity Confidence
Region 1.1	NRPS	183,660	228,084	SF2768	Medium
Region 1.2	terpene, NRPS-like, NRPS	248,336	323,998		
Region 1.3	NRPS, T1PKS	442,239	491,649	SGR PTMs	High
Region 1.4	terpene	522,026	548,584	hopene	High
Region 1.5	RiPP-like	627,710	637,925		
Region 1.6	RiPP-like	996,004	1,007,332		
Region 1.7	NI-siderophore	1,336,873	1,369,886		
Region 1.8	terpene	1,604,827	1,627,109	geosmin	High
Region 1.9	terpene-precursor	1,901,468	1,922,511		
Region 1.10	terpene	1,932,291	1,953,265	albaflavenone	High
Region 1.11	azole-containing-RiPP, RRE-containing	2,431,073	2,465,757		
Region 1.12	lassopeptide	3,231,737	3,254,225		
Region 1.13	NRPS	3,594,641	3,644,930		
Region 1.14	NRPS	3,680,239	3,742,474	dechlrocuracomycin	Low
Region 1.15	NRPS, LAP	3,934,786	4,043,436	surugamide A, D	High
Region 1.16	NRPS	4,499,022	4,543,416	dudomycin A	Low
Region 1.17	NI-siderophore	4,733,244	4,763,064	desferrioxamin B	High
Region 1.18	ectoine	5,626,152	5,636,550	ectoine	High
Region 1.19	NRPS	6,462,069	6,515,080	saframycins A,B	Low
Region 2.1	T1PKS, NRPS-like, NRPS, lanthipeptide-class-ii, terpene-precursor	100,865	371,911	candicidin	High
Region 2.2	T3PKS, RiPP-like	378,121	427,551	streptamidine	Medium
Region 2.3	terpene	486,161	514,760	isorenieratene	Medium

Table S5. Deduced putative functions of ORFs in **1**, **2** and candicidin biosynthetic gene cluster from region 2.1 of *Streptomyces* sp. AWH31-250.

ORFs	Size [bp] (aa)	Annotation based on BlastP Homologues from *Fsc* gene cluster (*Streptomyces* sp. FR-008)		
		Putative functions (Homologues)	Accession numbers	Query cover/ID
mohO	1218 (405)	FAD-dependent monooxygenase (***FscO***)	AAQ82549.1	100/99.26
mohP2	774 (257)	Aminotransferase class IV family protein (***PabC***)	AAQ82550.2	100/97.28
mohRI	696 (231)	PAS and helix-turn-helix domain-containing protein (***FscRI***)	AAQ82551.1	96/98.65
mohRII	2829 (942)	ATP-binding protein (AAA family ATPase) (***FscRII***)	AAQ82552.1	100/99.68
mohRIII	3042 (1013)	Helix-turn-helix transcriptional regulator (AAA family ATPase) (***FscRIII***)	AAQ82553.1	100/97.73
mohRIV	2913 (970)	Helix-turn-helix transcriptional regulator (AAA family ATPase) (***FscRIV***)	AAQ82554.1	100/98.56
mohMI	1377 (458)	Glycosyltransferase (***FscMI***)	AAQ82555.1	100/99.34
mohMII	1059 (352)	DegT/DrJ/EryC1/StrS family aminotransferase (***FscMII***)	AAQ82556.1	100/99.43
mohP	1182 (393)	Cytochrome P450 (***FscP***)	AAQ82557.1	100/100
mohFE	195 (64)	Ferredoxin (***FscFE***)	AAQ82558.1	100/96.88
mohTE	771 (256)	Thioesterase II family protein (***FscTE***)	AAQ82559.1	100/98.83
mohP1	2172 (723)	Aminodeoxychorismate synthase component I (***PabAB***)	AAQ82560.1	100/98.34
mohA	5229 (1742)	Type I polyketide synthase (A-ACP-KS-AT-ACP) (***FscA***)	AAQ82561.1	100/96.44
mohTI	1008 (335)	ATP-binding cassette domain-containing protein (***FscTI***)	AAQ82562.1	100/98.81
mohTII	843 (280)	ABC transporter permease (***FscTII***)	AAQ82563.1	100/99.16
mohC	31854 (10617)	SDR family NAD(P)-dependent oxidoreductase (KS-AT-DH-KR-ACP)*6 (***FscC***)	AAQ82564.1	100/97.28
mohB	16614 (5537)	Type I polyketide synthase (KS-AT-KR-CP-KS-AT-DH-ER-KR-CP-KS-AT-DH-KR-CP) (***FscB***)	AAQ82565.1	100/97.13
mohF	6150 (2049)	SDR family NAD(P)-dependent oxidoreductase (KS-AT-DH-KR-ACP) (***FscF***)	AAQ82566.1	100/98.15
mohD	26751 (8916)	Type I polyketide synthase (AT-DH-KR-ACP-KS-AT-KR-ACP-KS-AT-KR-ACP-KS-AT-KR-ACP-KS-AT-KRs-ACP-KS-AT-KR-ACP) (***FscD***)	AAQ82568.1	100/98.03
mohE	1800 (599)	Type I polyketide synthase, partial (KS) (***FscD-E)***	AAQ82567.1	100/59.20
mohMIII	1035 (344)	GDP-mannose 4,6-dehydratase (***FscMIII***)	WP_003946540.1	100/100
mohF1	759 (253)	Short-chain alcohol dehydrogenase family	SCD84506.1	100/99.60
mohF2	828 (276)	trans-aconitate 2-methyltransferase	WP_095709085.1	100/100
mohF3	762 (254)	Glycosyltransferase	AGI88817.1	100/99.61
mohF4	381 (127)	GNAT family acetyltransferase	WP_095730223.1	100/100
mohF5	411 (137)	Aspartate 1-decarboxylase	WP_095708454.1	100/100
mohF6	1068 (356)	Aldo/keto reductase	WP_359741129.1	100/100

Table S6. Summary of stereospecificity-determining motifs in β-module KRs: motifs highlighted in red denote primary motifs, while the remaining motifs serve as secondary support.

	G (1)	LDD (2)	R/Q (3)	K (4)	T (5)	D (6)	W (7)	H (8)	Y (9)	P (10)
**A0/A1**	G	xxx	x	K	x	**D**	**W**	Q	Y	A
**A0/A2**	G/A	xx**L**	x	K	x	**D**	**W**	**H**	Y	A
**B0/B1**	G	**LDD**	R	K	**T**	x	x	Q	Y	A
**B0/B2**	G/A	**LDD**	R/Q	K	**T**	x	x	Q/L	Y	**P**
**C0/C1**	G	xxx	x	**x**	x	x	x	x	**x**	x
**C0/C2**	No G	xxx	x	K	x	x	x	Q/H	**Y/Q**	A/P

Table S7. Amino acid sequences of ketoreductase (KR) of *mohB*, *mohC* and their homologs of *FscB* and *FscC*.

ORFs	Function	Sequence (aa)
mohB	KR1	TVLITGGTGALGAHVARSLAASGARHLVLTSRRGPGSPGAAELVAELEELGAHVTVADCDVADRDQLAGLLDSLPADLPLTGVVHAAGVLDDGVLDALTLDRFEAVLRPKTLGTAHLHELTRDHGVTLFVLFSSIVGVLGNAGQANYAAANAYLDAVAEQRRAEGLPVTSVAWGPWAD
	KR2	GTVLVTGGAGVLGGILARHLVTSHGMRHLVLTGRRGPDTPGAGELAAELRELGAEVTLAACDAADADALAAVLAAVPAEHPLTAVVHAAGVLDDGVLESLTPDRLRTVLAPKADAARHLHRLTRDLDLADLVLFSAGAGVFGNAGQANYAAANTYLDALAHRRRADGLPTTSLSWGLWAD
	KR3	GTVLITGGTGTLGALVARHLVTRHGIRHLLLTSRRGPDAPGAAALREELAVLGAETAIVACDVADREQLAGALDAVPAEHPLTAVVHTAGVLDDGTLDGLTPERVAHVLRPKADAAWHLHELTRDTKLTAFVMFSSYAGVAGGPGQANYAAANAFLDALAQHRRASGLPAHSLAWGLWED
mohC	KR1	GTVLLTGGTGGLGRIVARHLVVERGVRDLLLVSRSGAAADGAGQLVAELSEAGAHVTVEACDVSDPAAVAELVARHPVRAVVHSAGVLDDGTVESLTAERVTRVLRPKADAAWNLHEATRALDLEAFVVFSSMSGILGGPGQANYAAGNTFLDALVRHRRSLGLPATSLAWGPWT
	KR2	GTVLLTGGTGGLGRIVARHLVVERGVRDLLLVSRSGAAAEGVDAFTAELTGLGARVSVAACDLADRTALDALLAGVPADRPVRAVVHAAGVLDDGMAESLTAERVAAVLRPKVDAAWNLHEATRGLDLEAFVVFSSVAGTFGSAGQGAYAAGNVFLDALVEYRRALGLPGVSLVWGPWA
	KR3	VLITGGTGGLGRVLARHLVMTHGVRDLLLVSRSGATAEGAAELVTELTEAGAHVAVEACDAADADAVAGLVTRHGVRAVVHAAGVIDDATLASLTAERVAAVLRPKVDAAWNLHEATRDLGLEAFVVFSSVAGTFGSAGQGAYAAGNAFLDALVEYRRALGLPGVSLVWGPWA
	KR4	GAVLVTGGTGGLGAVMARYLVAERGVRDLVLVSRRGGDAPGAAELAAELREAGAAVEVVACDLSDRESVVELVGSLVSGRGLLAVVHAAGVGDNGLLGSLTLDRFDAVLGAKADAAWWLHEATAGVELAAFVLVSSAGGLVLTAGQGNYAAANVFLDALAARRRAGGLVATSMAFGFWD
	KR5	GAVLVTGGTGGLGAVMARYLVAERGVRDLVLVSRRGGDAPGAAELAAELREAGAAVEVVACDLSDRESVVELVGSLVSGRGLLAVVHAAGVGDNGLLGSLTLDRFDAVLGAKADAAWWLHEATAGVELAAFVLVSSAGGLVLTAGQGNYAAANVFLDALAARRRAGGLVATSMAFGFWD
	KR6	GTVLITGGTGGLGATVARHLVAEHGVRSLLLVSRRGPAAEGAGELAAALEESGARVTVAACDVGDRTAVDALLAEIPADRPLRAVVHAAGVLDDGVLDSLTPERLAAVLRPKADAAWHLHEATAGLPLDAFVLFSSVAGTLGSAGQANYAAGNAFLDALARFRRDRGLPAVSMAWGPWT
FscB	KR1	TVLITGGTGALGAHVARSLAASGARHLVLTSRRGPGSPGAVGLVAELEELGARVTVADCDVADRDQLARLLDSLPAGLPLTGVVHAAGVLDDGVLDALTLDRFEAVLRPKTLGTAHLHELTRDHDVTLFVLFSSIVGVLGNAGQANYAAANAYLDAVAEQRRAEGLPVTSVAWGPWAD
	KR2	GTVLVTGGAGVLGGILARHLVTRHGMRHLVLTGRRGPDTPGAGELAAELRELGAEVTLAACDAADADALAAVLAAVPAEHPLTAVVHAAGVLDDGVLESLTPDRLRTVLAPKADAARHLHRLTRDLDLTDLVLFSAGAGVFGNAGQANYAAANTYLDALAHRRRADGLPTTSLSWGLWAD
	KR3	GTVLITGGTGTLGALVARHLVTRHGVRHLLLTSRRGPGAPGAAALREELAALGAEAEIVACDVADREQLAGVLDAVPAEHPLTAVVHTAGVLDDGTLDGLTADRVAHVLRPKADAAWHLHELTRDTKLTAFVMFSSYAGVAGGPGQANYAAANAFLDALAQHRRASGLPAHSLAWGLWED
FscC	KR1	GTVLLTGGTGGLGRIVARHLVVERGVRDLLLVSRSGAAADGAGQLVAELSETGAHVTVEACDVSDPAAVAELVARHPVRAVVHSAGVLDDGTVESLTAERVTRVLRPKADAAWNLHEATRALDLEAFVVFSSMSGILGGPGQANYAAGNTFLDALVRHRRSLGLPATSLAWGPWT
	KR2	GTVLLTGGTGGLGRIVARHLVVERGVRDLLLVSRSGAAAEGVDAFTAELTGLGARVSVAACDLADRTALDALLAGVPADRPVRAVVHAAGVLDDGMAESLTAERVAAVLRPKVDAAWNLHEATRGLDLEAFVVFSSVAGTFGSAGQGAYAAGNVFLDALVEYRRSLGLPGVSLVWGPWA
	KR3	VLITGGTGGLGRVLARHLVMTHGVRDLLLVSRSGANAEGAGELVTELTEAGAHVAVEACDAADADAVAGLVTRHGVRAVVHAAGVIDDATLASLTAERVAAVLRPKVDAAWNLHEATRDLGLEAFVVFSSVAGTFGSAGQGAYAAGNVFLDALVEYRRALGLPGVSLVWGPWA
	KR4	GAVLVTGGTGGLGAVMARYLVAERGVRDLVLVSRRGGDAPGAAELAGELREAGAAVEVVACDLSDRESVVGLVESLVAGRGLRAVVHAAGVGGGGLVGTLSSDRFDAVLGAKADAAWWLHEATAGVELAAFVLVSSAGGLVLTAGQGNYAAANVFLDALASRRRAEGLVATSMAFGFWD
	KR5	GAVLVTGGTGGLGAVMARYLVAERGVRDLVLVSRRGGDAPGAAELAGELREAGAAVEVVACDLSDRESVVDLVGSLVSGRGLLAVVHAAGVGDNGLLGSLTPDRFDAVLGAKADAAWWLHEATAGVELAAFVLVSSAGGLVLTAGQGNYAAANVFLDALASRRRAEGLVATSMAFGFWD
	KR6	GTVLITGGTGGLGATVARHLVAEHGVRSLLLVSRRGPAAEGAGELAAALEESGAHVTVAACDVGDRTAVDALLAEIPADRPLRAVIHAAGVLDDGVLDSLTPERLAAVLRPKADAAWHLHEATEGLTLDAFVLFSSVAGTLGSAGQANYAAGNAFLDALARHRRDRGLPAVSMAWGPWT

Table S8. Amino acid sequences of representative *β*-module ketoreductase (KR) subtypes.

Type	Name	Sequence (aa)
A1	*Ery2*	GTILVTGGTAGLGAEVARWLAGRGAEHLALVSRRGPDTEGVGDLTAELTRLGARVSVHACDVSSREPVRELVHGLIEQGDVVRGVVHAAGLPQQVAINDMDEAAFDEVVAAKAGGAVHLDELCSDAELFLLFSSGAGVWGSARQGAYAAGNAFLDAFARHRRGRGLPATSVAWGLWA
	Meg6	GTALVTGGTGALGGHVARYLARSGVTDLVLLSRSGPDAPGAAELAAELADLGAEPRVEACDVTDGPRLRALVQELREQDRPVRIVVHTAGVPDSRPLDRIDELESVSAAKVTGARLLDELCPDADTFVLFSSGAGVWGSANLGAYAAANAYLDALAHRRRQAGRAATSVAWGAWA
	Sor6	GTILITGGTGAIGAHVARWLARKGAEHLVLISRRGAQAEGAVELHAELTALGARVTFAACDVADRSAVATLLEQLDAGGPQVSAVFHAGGIEPHAPLAATSMEDLAEVVSGKVQGARHLHDLLGSRPLDAFVLFSSGAVVWGGGQQGGYAAANAFLDALAEQRRSLGLTATSVAWGVWG
A2	*Con5*	GTVLVTGGTGGVGAHLARWLAKGGAEHLVLVGRRGADTPGAADLTAELTALGTRVTVAACDVADRDRLAELVAGLEQDGPPIRTVIHAAGTGLLVPLSDTDPDEFADTLYAKVAGAENLDAVFDRDDLDTFVLFSSISGVWGSGDHGAYAAANAHLDALADRRRARGRTATSVVWGIWD
	Can13	GTVLVTGGTGTLGPHLARWLADQGARDLVLTSRRGADAPGAAELAAELGERGCRVTVAACDVTDREAVAALLDGLTAEGRTVRSVFHTAAVIELQSIEETSLDAFAKVVHAKTAGAAHLDELLDDDQLDAFVLYSSTAGMWGSGRHAAYVAANAHVNALAEHRCARGAHGTAVSWGIWAD
	Ela4	GTVLLTGGTGGVGAQIARRLAQAGAEHLVLTSRRGPDAPGADKLKAELTELGAKVTVAACDVADRAALEALVRKVEAEGPPIRSVLHIAGAGVLVPLADTDLAEFADTAEAKVAGAANLDALFDRDTLDSFVLFSSISAVWGSGEHGAYAAANAYLDGLAEHRRARGLTATSVVWGIWS
	Nys1	GTTLITGGSGTLAPQLTRWLAARGAEHVVLVSRRGAHAPGAPELAAELAESGTEVTPVACDITDRDAVAALLADLKADGRTVRTVLHAAATIELSALADTTVAEFADVVHAKVTGAQILDELLDDEELDDFVLYSSTAGMWGSGVHAAYVAGNAYLSALAERRRARGQRATSVHWGKWP
B1	*Baf1*	GTVLVTGATGGVGSHTARWLAGQGAERLLLVSRRGADAPGTDALRAELTALGAEVAFAACDVADPAALAALVAGIPARYPLSAVVHAAGVLDDGVLDALDPARLAVVLRAKLTAARNLHEATADLGLSAFVVFSSVMGVVGNAGQGNYAAANAAVDALVAARRAAGLPGTSVAWGAWA
	Baf2	GTVLITGGTGGLGAQIARWLARTGTAHLLLTSRRGAQAPGADELLAELRGLGATATAVACDVADRDALAELLAGIPAERPLRAVLHTAGVLDDGVIDAITPERAAGVLRPKLDGARNLDELTRELDLTAFVLFSSLAGTLGGTGQGSYAAANAYLDALARHRRDLGLPGTSVAWGLWG
B2	*Lan1*	GTALVTGGTGGIGRNVARWLAQHGAPHIVLVSRSGPEAPGAGELAAELRALGSAVTLAACDVTDREALRSLLAALPADQPLTSVFHTAATLDDGTLDTLTGERIEQAGRAKTLGARNLHELTADAPLTAFVLFSSFASAFGAPGLGCYAPGNAYLDGLARRRRADGLPATSVAWGTWA
	Meg1	GTVLVTGGTGGVGRHVARWLARQGTPCLVLASRRGPDADGVEELLTELADLGTRATVTACDVTDREQLRALLATVDDEHPLSAVFHVAATLDDGTVETLTGDRIERANRAKVLGARNLHELTRDADLDAFVLFSSSTAAFGAPGLGGYVPGNAYLDGLAQQRRSEGLPATSVAWGTWA
C1	*Oli14*	GTVLITGGTGGIGAHLARWLAKEGAERLLLVSRSGERAEGAAELAAELGGLGAEVTFAACDVTDRDALAHVIAGIPAEHPLTAVFHTAGVAGHGPLADLDVTDLDTQTAARIAGARHLDELTAELGVELEAFVVFSSGAAVWGSGSNGANAAAGGYLDGLVRLRRARGVAGTSVSWGGWQ
C2	*Meg3*	GTVLVTGAAGPVGGRLARWLAERGATRLVLPGAHPGEELLTAIRAAGATAVVCEPEAEALRTAIGGELPTALVHAETLTNFAGVADADPEDFAATVAAKTALPTVLAEVLGDHRLEREVYCSSVAGVWGGVGMAAYAAGSAYLDALVEHRRARGHASASVAWTPWA
	Lan3	GTVLLTGADSPIGARLARWAADSGADHLLLVGEADEELLNALRERGTTATRCAADEDALRAAVEAAPHDISTVVHAATRTEFGPVLATDPEDFAATLQAKTGLALTLAAVLDGRPVEREIHCSSVAGVWGGAGMAGYAAGSACLDAFAAHRRAQGHPSTAVAFSPWA

* Baf: Bafilomycin, Can: Candicidin, Con: Concanamycin, Ela: Elaiophylin, Ery: erythromycin, Lan: Lankamycin, Meg: Megalomycin, Nys: Nystatin, Oli: Oligomycin, Sor: Soraphen

Table S9. Comparative sequence alignment of ketoreductase (KR) domains from *mohB*, *mohC* and their homologs *FscB* and *FscC*.

ORFs	Size (aa)	Annotation based on BlastP<br>Homologues from *Fsc* gene cluster (*Streptomyces* sp. FR-008)	
		**Putative functions (Homologues)**	**Query cover/ID**
*mohB*_KR1	178	Beta-ketoacyl reductase **(*FscB*_KR1)**	100/96.63
*mohB*_KR2	180	SDR family NAD(P)-dependent oxidoreductase, partial **(*FscB*_KR2)**	100/98.89
*mohB*_KR3	180	Beta-ketoacyl reductase **(*FscB*_KR3)**	100/95.56
*mohC*_KR1	175	Beta-ketoacyl reductase **(*FscC*_KR1)**	100/99.43
*mohC*_KR2	179	Type I polyketide synthase **(*FscC*_KR2)**	100/99.44
*mohC*_KR3	173	Type I polyketide synthase, partial **(*FscC*_KR3)**	100/98.27
*mohC*_KR4	179	Beta-ketoacyl reductase, partial **(*FscC*_KR4)**	100/92.17
*mohC*_KR5	179	Beta-ketoacyl reductase, partial **(*FscC*_KR5)**	100/97.21
*mohC*_KR6	179	Type I polyketide synthase, partial **(*FscC*_KR6)**	100/97.21

Table S10. Results of antibacterial assay of **1** and **2**.

Sample	IC_{50} (μM)					
	Gram positive			Gram negative		
	S. aureus	*E. faecalis*	*E. faecium*	*K. pneumonia*	*S. enterica*	*E. coli*
Mohangic acid H (**1**)	>128	>128	>128	>128	>128	>128
Mohangiol (**2**)	>128	>128	>128	>128	>128	>128
Ampicillin *	0.063	0.5	0.5	64	0.13	8
Tetracycline *	ND	ND	ND	0.25	ND	ND

* Ampicillin and tetracycline were used as a positive control of antibacterial assay. ND: not determined

Table S11. Results of antifungal assay of **1** and **2**.

Sample	IC_{50} (μM)			
	C. albicans	*A. fumigatus*	*T. rubrum*	*T. mentagrophytes*
Mohangic acid H (**1**)	>128	>128	>128	>128
Mohangiol (**2**)	>128	>128	>128	>128
Amphotericin B *	1	4	4	4

* Amphotericin B was used as a positive control of antifungal assay.
